# Renal Recovery Patterns and Long‐Term Kidney Function Decline in Pulmonary Hypertension

**DOI:** 10.1002/pul2.70336

**Published:** 2026-06-04

**Authors:** Lukas Hintz, Anastasios Stampouloglou, Janani Rangaswami, Kevin Brian Lo, Werner Seeger, Hossein‐Ardeschir Ghofrani, Henning Gall, Khodr Tello, Faeq Husain‐Syed

**Affiliations:** ^1^ Department of Internal Medicine II University Hospital Giessen and Marburg, Justus‐Liebig‐University Giessen Giessen Hessen Germany; ^2^ Department of Pulmonology Evangelische Lungenklinik Berlin Berlin Germany; ^3^ VA Medical Center Washington DC USA; ^4^ George Washington University School of Medicine Washington DC USA; ^5^ Department of Cardiology Brigham and Women's Hospital, Harvard Medical School Boston MA USA; ^6^ Department of Internal Medicine Universities of Giessen and Marburg Lung Center (UGMLC), Institute for Lung Health (ILH), Cardio‐Pulmonary Institute (CPI), Member of the German Center for Lung Research (DZL) Giessen Germany; ^7^ Department of Pulmonology Kerckhoff‐Klinik Bad Nauheim Hessen Germany; ^8^ Department of Pulmonology Hochtaunus‐Kliniken, Krankenhaus Usingen Usingen Hessen Germany; ^9^ Department of Pulmonology and Ventilator Medicine Krankenhaus Nordwest Frankfurt am Main Hessen Germany

## Abstract

Pulmonary hypertension (PH) is frequently accompanied by a decline in kidney function. In this retrospective cohort, worsening PH was associated with long‐term eGFR decline only when accompanied by transition from acute kidney injury (AKI) to acute kidney disease (AKD), defined as persistent kidney dysfunction beyond 7 days after AKI onset, whereas early AKI reversal was not. These findings suggest that renal recovery patterns, rather than transient changes in kidney function, may determine longitudinal kidney outcomes in PH and warrant further prospective investigation.

Pulmonary hypertension (PH) is a progressive disease of the pulmonary vasculature and is frequently accompanied by abnormal kidney function [1]. While cardiorenal interactions have been extensively studied in left‐sided heart failure (HF) [2], they are less well‐characterized in PH, where kidney disease is predominantly driven by right‐sided HF physiology [3]. In this context, elevated right atrial pressure (RAP) leads to renal venous congestion, increased renal interstitial pressure, and a reduction in effective renal perfusion pressure—key hemodynamic derangements underlying congestive nephropathy [4].

Episodes of PH‐related clinical worsening may precipitate worsening renal function (WRF), frequently fulfilling KDIGO (Kidney Disease: Improving Global Outcomes) criteria for acute kidney injury (AKI) [5]. In the contemporary kidney disease framework, incomplete recovery from AKI beyond the acute phase is referred to as acute kidney disease (AKD), describing persistent kidney dysfunction lasting from 7 to 90 days after the initial insult [6]. In cardiorenal syndrome, WRF episodes likely represent acute‐on‐chronic congestive nephropathy, in which transient worsening of venous congestion and reduced renal perfusion pressure drive rises in serum creatinine [4]. However, whether these creatinine increases reflect reversible, hemodynamically mediated reductions in GFR or structural kidney injury with incomplete recovery and adverse renal trajectories remains unknown in PH. The renal consequences of the AKI–AKD transition have not been systematically examined in this population.

This retrospective single‐center cohort study included adult patients with PH Groups 1–4 (i.e., pulmonary arterial hypertension (PAH), PH due to left heart disease (PH‐LHD), PH due to chronic lung disease and/or hypoxia, and chronic thromboembolic PH) undergoing right heart catheterization (RHC) at a tertiary German center between February 2008 and December 2016. The study period was chosen to allow sufficient longitudinal follow‐up to capture clinically meaningful changes in kidney function while ensuring data completeness and consistency within the Giessen PH Registry [7]. PH was defined as a mean pulmonary arterial pressure ≥ 20 mmHg at rest [8]. Exclusion criteria included absence of PH, exercise‐induced PH, multifactorial or indeterminate PH in which no single underlying mechanism could be reliably classified, CKD stage 5, previous organ transplantation, and absence of serum creatinine measured on the day of RHC. The study was approved by the Ethics Committee of Justus Liebig University Giessen (approval number 238/16) and registered at ClinicalTrials.gov (Identifier: NCT03045614). Due to the retrospective nature of the study, the requirement for informed consent was waived by the Ethics Committee.

Worsening PH was defined as unplanned hospitalizations for PH‐related clinical deterioration, identified through a systematic review of medical records. WRF was defined as a rise in serum creatinine ≥ 0.3 mg/dL during hospitalization in accordance with KDIGO AKI criteria [5]. AKI trajectories were operationalized as early reversal (AKI recovery within 7 days of onset) or persistence beyond 7 days, consistent with the KDIGO‐based AKD definition [9].

Continuous variables were expressed as the mean (± standard deviation) or median (interquartile range). Categorical variables were expressed as the number of participants (%). Patient characteristics were compared within subgroups using ANOVA, Kruskal–Wallis test or pairwise Chi‐squared test, as appropriate. Longitudinal renal outcomes were evaluated as the absolute change in eGFR over 3 years, calculated using the CKD‐EPI 2009 creatinine‐based equation. The 3‐year eGFR value was required to be sustained, defined as at least two serum creatinine measurements obtained ≥ 90 days apart and not during hospitalization, and was confirmed by a nephrologist (FH‐S) not involved in data collection. Patients who underwent lung transplantation or pulmonary thromboendarterectomy were excluded from follow‐up analyses. Missing data were handled by complete case analysis. The proportion of missing data was low and assumed to be missing at random; therefore, no imputation was performed. A two‐sided *p*‐value < 0.05 was considered statistically significant. Statistical analyses were performed using SPSS Statistics, version 26 (IBM Corp., Armonk, NY, USA).

A total of 824 patients with pulmonary hypertension were included (Figure S1). The mean age was 66 years, 54% were female. Most patients had advanced disease, with 81% in WHO functional class III/IV, and a high burden of comorbidities, including hypertension and diabetes mellitus, particularly among patients with PH‐LHD (Table S1). CKD was present in 38.4% of patients and varied by PH etiology, being highest in PH‐LHD (52.0%) and lowest in PAH (25.4%).

Longitudinal kidney function trajectories were available for 316 (38.4%) patients and differed across PH groups, with higher availability in PAH (96/169, 56.8%) and lower availability in PH‐LHD (73/271, 26.9%), while CLD‐PH (87/228, 38.2%) and CTEPH (60/156, 38.5%) showed intermediate follow‐up rates. Over 3 years, patients with PH‐LHD exhibited the greatest decline in eGFR, whereas patients with PAH showed comparatively preserved kidney function (*p* < 0.001; Figure 1A and Table S2).

**Figure 1:**
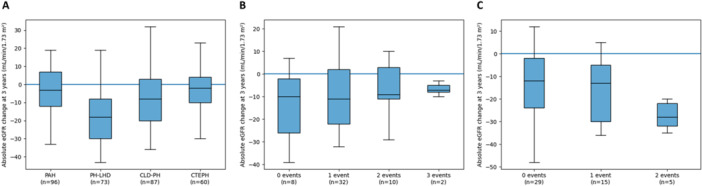
Longitudinal change in eGFR according to PH group and worsening PH events. Longitudinal eGFR decline by PH Group (A), by number of worsening PH events accompanied with early reversal of AKI (B), and by number of worsening PH events accompanied with AKI‐AKD transition (C). AKI, acute kidney injury; AKD, acute kidney disease; CLD‐PH, pulmonary hypertension due to chronic lung disease and/or hypoxia; CTEPH, chronic thromboembolic pulmonary hypertension; eGFR, estimated glomerular filtration rate; PAH, pulmonary arterial hypertension; PH, pulmonary hypertension; PH‐LHD, pulmonary hypertension due to left heart disease.

Worsening PH events occurred across all PH subgroups in the overall cohort, with similar proportions of patients with ≥ 1 event in PAH and PH‐LHD, and lower rates in CLD‐PH and CTEPH (Table S3). Among patients experiencing worsening PH with early reversal of AKI, eGFR decline did not show a clear dose–response relationship with the number of events (*p* = 0.80; Figure 1B and Table S4). In contrast, worsening PH accompanied by AKI‐AKD transition was associated with a stepwise decline in eGFR, with increasing numbers of worsening PH events corresponding to progressively greater long‐term kidney function loss (*p* = 0.028; Figure 1C and Table S5).

In this retrospective single‐center analysis of patients with PH, worsening PH was associated with subsequent long‐term decline in eGFR when accompanied by transition from AKI to AKD, whereas episodes accompanied by early reversal of AKI were not associated with significant long‐term kidney function decline. These findings suggest that not all episodes of WRF in PH may carry the same prognostic renal implications. This distinction is consistent with the concept that transient rises in serum creatinine may reflect predominantly functional, hemodynamically mediated reductions in GFR related to venous congestion and right‐sided HF, rather than sustained structural kidney injury. Similar observations have been reported in left‐sided HF, where WRF during effective decongestion is often reversible and not necessarily associated with acute tubular damage or adverse outcomes [10, 11]. Our data suggest that a comparable paradigm may apply in PH. Importantly, progression from AKI to AKD appears to identify a higher‐risk phenotype characterized by persistent kidney dysfunction and adverse long‐term renal trajectories. This supports the notion that renal recovery patterns provide clinically relevant information beyond creatinine changes alone and may improve risk stratification in PH. While differences in renal trajectories across PH subgroups likely reflect underlying comorbidities and disease‐specific mechanisms, particularly in PH‐LHD, the association between AKI recovery patterns and long‐term kidney outcomes was observed across the overall cohort, suggesting that renal recovery phenotype may represent a unifying pathophysiologic signal across heterogeneous PH phenotypes.

Interpretation is limited by the retrospective single‐center design, precluding causal inference. In addition, the absence of AKI biomarkers, lack of longitudinal adjustment for PH‐specific therapies, and PH‐specific therapy data recorded at the time of RHC, which do not reflect subsequent treatment escalation or longitudinal adherence to guideline‐directed therapy, and missing data on albuminuria—precluding full CKD classification according to KDIGO criteria—limit mechanistic insight. Furthermore, longitudinal kidney function trajectories were available in only 38% of the cohort, introducing potential selection bias. Follow‐up data were more frequently available in PAH and less frequently in PH‐LHD, likely reflecting referral and follow‐up patterns in a specialized PH center. Notably, PH‐LHD (i.e., the subgroup with the greatest eGFR decline) was underrepresented, suggesting that the observed associations may underestimate the true magnitude of kidney function loss in this PH group. Given the limited sample size in several subgroups, particularly among patients with multiple worsening PH events, multivariable adjustment was not performed to avoid model overfitting and unstable estimates; therefore, findings should be interpreted as descriptive and hypothesis‐generating. Nevertheless, the consistent divergence in renal trajectories according to AKI recovery patterns supports the concept that recovery phenotype, rather than AKI occurrence alone, is associated with long‐term kidney outcomes and suggests that distinguishing hemodynamically mediated from sustained structural kidney injury may be clinically relevant in patients with PH. These findings warrant prospective investigation in biomarker‐informed studies designed to differentiate functional from structural kidney injury.

In conclusion, CKD was present in approximately one‐third of patients with PH. Worsening PH, particularly when accompanied by AKI‐AKD transition, may identify a subgroup of patients at risk for accelerated long‐term kidney function decline.

## Author Contributions

The authors share responsibility for study design, data collection, data analysis, and data interpretation, as well as preparation, review, and approval of the manuscript. F‐HS is the senior authorship and takes final responsibility for the decision to submit for publication. Study concept and design: AT, HG, and FH‐S. Literature research and clinical advice: LH, AT, JR, WS, H‐AG, HG, KT, and FH‐S. Acquisition, analysis, or interpretation of data: LH, AT, JR, WS, H‐AG, HG, KT, and FH‐S. Drafting of the manuscript: LH, AT, and FH‐S. Critical revision of the manuscript for important intellectual content: LH, AT, JR, WS, H‐AG, HG, KT, and FH‐S. Statistical analysis: HG and FH‐S. Study supervision: HG and FH‐S.

## Funding

The authors have nothing to report.

## Ethics Statement

The study was approved by the Ethics Committee of Justus Liebig University Giessen (approval number 238/16).

## Conflicts of Interest

Janani Rangaswami discloses consulting for Boehringer Ingelheim, Bayer, and Procyrion (Aortix) outside the submitted work. Werner Seeger discloses grants from the German Research Foundation and non‐financial support from the University of Giessen during the conduct of the study, and personal fees from Pfizer and Bayer Pharma AG outside the submitted work. Hossein‐Ardeschir Ghofrani discloses grants from the German Research Foundation and non‐financial support from the University of Giessen during the conduct of the study, and personal fees from Bayer, Actelion, Pfizer, Merck, GSK, and Takeda, grants and personal fees from Novartis, Bayer HealthCare, and Encysive/Pfizer, and grants from Aires, the German Research Foundation, Excellence Cluster Cardiopulmonary Research, and the German Ministry for Education and Research outside the submitted work. Khodr Tello discloses grants from the German Research Foundation and non‐financial support from the University of Giessen during the conduct of the study, and personal fees from Actelion and Bayer outside the submitted work. Henning Gall reports grants from the German Research Foundation and non‐financial support from the University of Giessen during the conduct of the study, and personal fees from Actelion, AstraZeneca, Bayer, BMS, GSK, Janssen‐Cilag, Lilly, MSD, Novartis, OMT, Pfizer, GossamerBio, and United Therapeutics outside the submitted work. The remaining authors declare no conflict of interest.

## Supporting information


**Figure S1:** Study flow diagram.
**Table S1:** Baseline characteristics of the cohort.
**Table S2:** Absolute change in eGFR at 3 years by PH group.
**Table S3:** Distribution of PH decompensation events across the overall cohort.
**Table S4:** Absolute change in eGFR at 3 years according to number of PH decompensation events with early reversal of AKI.
**Table S5:** Absolute change in eGFR at 3 years according to number of PH decompensation events with AKI‐AKD transition.

## Data Availability

The data that support the findings of this study are available from the corresponding author, FH‐S, upon reasonable request.
