# Supplementary material for: Renal Recovery Patterns and Long‐Term Kidney Function Decline in Pulmonary Hypertension
Source: Pulm Circ. 2026 Jun 4;16(2):e70336. doi: 10.1002/pul2.70336 (PMC13347916; doi:10.1002/pul2.70336)
Supplement: Supplementary file 1 — Figure S1: Study flow diagram. Table S1: Baseline characteristics of the cohort. Table S2: Absolute change in eGFR at 3 years by PH group. Table S3: Distribution of PH decompensation events across the overall cohort. Table S4: Absolute change in eGFR at 3 years according to number of PH decompensation events with early reversal of AKI. Table S5: Absolute change in eGFR at 3 years according to number of PH decompensation events with AKI‐AKD transition. [file PUL2-16-e70336-s001.docx]

**SUPPLEMENTARY APPENDIX**

**Renal Recovery Patterns and Long-Term Kidney Function Decline in Pulmonary Hypertension**

Lukas Hintz, MD^#^, Anastasios Stampouloglou, MD^#^, Janani Rangaswami, MD, FACP, Kevin Bryan Lo, MD, MS, Werner Seeger, MD, Hossein-Ardeschir Ghofrani, MD, Henning Gall, MD, PhD, Khodr Tello, MD, Faeq Husain-Syed, MD

^#^Joint first authors

This supplementary material has been provided by the authors to give readers additional information about their work.

**TABLE OF CONTENTS**

[**SUPPLEMENTAL TABLES** 1](#_Toc227495508)

[Table S1. Baseline characteristics of the cohort 1](#_Toc227495509)

[Table S2. Absolute change in eGFR at 3 years by PH group 3](#_Toc227495510)

[Table S3. Distribution of PH decompensation events across the overall cohort 4](#_Toc227495511)

[Table S4. Absolute change in eGFR at 3 years according to number of PH decompensation events with early reversal of AKI 5](#_Toc227495512)

[Table S5. Absolute change in eGFR at 3 years according to number of PH decompensation events with AKI–AKD transition 6](#_Toc227495513)

[**SUPPLEMENTAL FIGURES** 7](#_Toc227495514)

[Figure S1. Study flow diagram 7](#_Toc227495515)

# **SUPPLEMENTAL TABLES**

## **Table S1. Baseline characteristics of the cohort**

| **Parameter** | **All patients**  **(n = 824)** | **PAH**  **(n = 169)** | **PH-LHD**  **(n = 271)** | **CLD-PH**  **(n = 228)** | **CTEPH**  **(n = 156)** | **P-Value** |
| --- | --- | --- | --- | --- | --- | --- |
| Age, years | 69.0 [57–76] | 55.5 [42–68] | 73 [67–78] | 68 [61–75] | 69 [56–76] | <0.001 |
| Female gender, % | 448 (54.4%) | 108 (63.9%) | 189 (69.4%) | 89 (39.0%) | 85 (54.5%) | <0.001 |
| Weight, kg | 81.2 ± 19.7 | 74 ± 17 | 84 ± 20 | 82 ± 21 | 83 ± 19 | <0.001 |
| NYHA functional class III–IV, % | 548 (67%) | 103 (61%) | 179 (66%) | 157 (69%) | 109 (70%) | <0.001 |
| 6MWD, m | 304 ± 130 | 349 ± 129 | 300 ± 122 | 251 ± 119 | 333 ± 130 | <0.001 |
| Hypertension, % | 551 (67%) | 74 (44%) | 232 (86%) | 151 (67%) | 94 (61%) | <0.001 |
| Diabetes, % | 206 (25%) | 11 (17%) | 45 (34%) | 29 (29%) | 11 (12%) | <0.001 |
| PH-specific therapy, % ^1^ | 354 (43%) | 93 (55%) | 52 (19%) | 129 (57%) | 80 (51%) | <0.001 |
| RAS inhibition, % | 489 (59%) | 64 (38%) | 227 (84%) | 121 (54%) | 77 (50%) | <0.001 |
| Loop diuretic, % | 543 (66%) | 88 (52%) | 235 (87%) | 135 (60%) | 85 (55%) | <0.001 |
| Thiazide diuretic, % | 330 (40%) | 45 (27%) | 144 (53%) | 82 (36%) | 59 (38%) | <0.001 |
| Combined diuretics, % ^2^ | 246 (30%) | 31 (18%) | 123 (45%) | 56 (25%) | 36 (23%) | <0.001 |
| LVEF, % | 61 ± 11 | 66 ± 8 | 57 ± 13 | 63 ± 8 | 63 ± 8 | <0.001 |
| mPAP, mmHg | 38 ± 10 | 44 ± 13 | 35 ± 8 | 34 ± 8 | 40 ± 10 | <0.001 |
| PAWP, mmHg | 12 ± 6 | 8 ± 3 | 19 ± 5 | 9 ± 3 | 9 ± 5 | <0.001 |
| RAP, mmHg | 7 ± 5 | 5 ± 4 | 9 ± 5 | 5 ± 4 | 7 ± 5 | <0.001 |
| PVR, dyn × s/cm^5^ | 385 [249–614] | 606 [380–841] | 242 [171–373] | 379 [288–528] | 569 [367–800] | <0.001 |
| CI, L/min/m^2^ | 2.4 ± 0.7 | 2.7 ± 0.9 | 2.5 ± 0.7 | 2.6 ± 0.7 | 2.3 ± 0.6 | <0.001 |
| Serum creatinine, mg/dL | 1.1 ± 0.4 | 1.0 ± 0.3 | 1.2 ± 0.4 | 1.0 ± 0.4 | 1.1 ± 0.3 | 0.001 |
| eGFR, mL/min/1.73 m^2 3^ | 69 ± 24 | 80 ± 26 | 60 ± 22 | 72 ± 22 | 67 ± 21 | <0.001 |
| eGFR <60 mL/min/1.73 m^2^, % ^3^ | 309 (38%) | 43 (25%) | 139 (51%) | 73 (32%) | 54 (35%) | <0.001 |
| BNP, pg/mL | 160 [65–344] | 141 [53–284] | 212 [116–405] | 104 [50–241] | 166 [59–407] | <0.001 |

Data are presented as mean ± standard deviation, median [interquartile range], or number (%), as appropriate. P values refer to comparisons across PH subgroups.

^1^ PH-specific therapy data reflect treatment status at the time of right heart catheterization and do not capture subsequent treatment escalation or longitudinal adherence to guideline-directed therapy.

^2^ Combined diuretics indicate concurrent use of loop and thiazide diuretics.

^3^ eGFR was calculated using the 2009 Chronic Kidney Disease Epidemiology Collaboration (CKD-EPI) creatinine-based equation.

6MWD, 6-minute walk distance; BNP, B-type natriuretic peptide; CI, cardiac index; CLD-PH, pulmonary hypertension due to chronic lung disease and/or hypoxia; CTEPH, chronic thromboembolic pulmonary hypertension; eGFR, estimated glomerular filtration rate; LVEF, left ventricular ejection fraction; mPAP, mean pulmonary arterial pressure; NYHA, New York Heart Association; PAH, pulmonary arterial hypertension; PAWP, pulmonary arterial wedge pressure; PH-LHD, pulmonary hypertension due to left heart disease; PVR, pulmonary vascular resistance; RAP, right atrial pressure; RAS, renin–angiotensin system.

## **Table S2. Absolute change in eGFR at 3 years by PH group**

| PH Group | Absolute eGFR change at 3 years, mL/min/1.73 m² (median [IQR]) | **N** |
| --- | --- | --- |
| PAH | −4 [−12 to 7] | 96 |
| PH-LHD | −18 [−30 to −8] | 73 |
| CLD-PH | −8 [−20 to 3] | 87 |
| CTEPH | −2 [−10 to 4] | 60 |

**P value (across groups):** <0.001.

eGFR, estimated glomerular filtration rate; PAH, pulmonary arterial hypertension; PH-LHD, pulmonary hypertension due to left heart disease; CLD-PH, pulmonary hypertension due to chronic lung disease and/or hypoxia; CTEPH, chronic thromboembolic pulmonary hypertension.

## **Table S3. Distribution of PH decompensation events across the overall cohort**

| **PH group** | **Total (n)** | **≥1 event, n (%)** | **Total number of events** |
| --- | --- | --- | --- |
| PAH | 163 | 26 (16.0%) | 30 |
| PH-LHD | 251 | 41 (16.3%) | 54 |
| CLD-PH | 213 | 25 (11.7%) | 36 |
| CTEPH | 148 | 11 (7.4%) | 15 |

Data refer to the overall study cohort. Decompensation events were defined as PH-related hospitalizations for clinical worsening. Percentages represent the proportion of patients with ≥1 event within each PH subgroup.

PAH, pulmonary arterial hypertension; PH, pulmonary hypertension; PH-LHD, pulmonary hypertension due to left heart disease; CLD-PH, pulmonary hypertension due to chronic lung disease and/or hypoxia; CTEPH, chronic thromboembolic pulmonary hypertension.

## **Table S4. Absolute change in eGFR at 3 years according to number of PH decompensation events with early reversal of AKI**

| Number of worsening PH events | Absolute eGFR change at 3 years, mL/min/1.73 m² (median [IQR]) | **N** |
| --- | --- | --- |
| 0 events | −10 [−26 to −2] | 8 |
| 1 event | −11 [−22 to 2] | 32 |
| 2 events | −9 [−11 to 3] | 10 |
| 3 events | −7 [−8 to −5] | 2 |

**P value (trend across groups):**0.80**.**

eGFR, estimated glomerular filtration rate; AKI, acute kidney injury; PH, pulmonary hypertension.

## **Table S5. Absolute change in eGFR at 3 years according to number of PH decompensation events with AKI–AKD transition**

| Number of worsening PH events | Absolute eGFR change at 3 years, mL/min/1.73 m² (median [IQR]) | **N** |
| --- | --- | --- |
| 0 events | −12 [−24 to −2] | 29 |
| 1 event | −13 [−30 to −5] | 15 |
| 2 events | −28 [−32 to −22] | 5 |

**P value (trend across groups):**0.028**.**

eGFR, estimated glomerular filtration rate; AKI, acute kidney injury; PH, pulmonary hypertension.

# **SUPPLEMENTAL FIGURES**

## **Figure S1. Study flow diagram**

Flow diagram of patient selection.

CKD, chronic kidney disease; RHC, right heart catheterization; PH, pulmonary hypertension.
